# Testing population-based performance measures identifies gaps in juvenile idiopathic arthritis (JIA) care

**DOI:** 10.1186/s12913-019-4379-4

**Published:** 2019-08-14

**Authors:** Claire E.H. Barber, Lisa M. Lix, Diane Lacaille, Deborah A. Marshall, Kristine Kroeker, Susanne Benseler, Marinka Twilt, Heinrike Schmeling, Cheryl Barnabe, Glen S. Hazlewood, Vivian Bykerk, Joanne Homik, J. Carter Thorne, Jennifer Burt, Dianne Mosher, Steven Katz, Natalie J. Shiff

**Affiliations:** 1 Department of Medicine, Division of Rheumatology, Cumming School of Medicine, University of Calgary, 3330 Hospital Drive NW, Calgary, AB T2N 4N1 Canada; 2 University of Manitoba, S113-750 Bannatyne Ave, Winnipeg, MB R3E 0W3 Canada; 3 Arthritis Research Canada, 5591 No. 3 Road, Richmond, BC V6X 2C7 Canada; 4 Department of Community Health Sciences, Cumming School of Medicine, University of Calgary, 3280 Hospital Drive NW, Calgary, AB T2N 4Z6 Canada; 5 George & Fay Yee Centre for Healthcare Innovation, 3rd floor, 753 McDermot Ave, Winnipeg, MB R3E 0T6 Canada; 6 Alberta Children’s Hospital, 28 Oki Drive, Calgary, T3B 6A8 AB Canada; 7 Hospital for Special Surgery, 535 E 70th St, New York, NY USA; 8 3A Medicine Clinic, Third Floor, Edmonton Clinic, 11400 University Ave, Edmonton, AB T6G 1Z1 Canada; 9 43 Lundy’s Lane, Newmarket, ON L3Y 3RY Canada; 10 Rheumatology Services, St. Clare’s Mercy Hospital, 154 LeMarchant Road, St. John’s, NL A1C 5B8 Canada; 11 Third Floor, Edmonton Clinic, 11400 University Ave, Edmonton, AB T6G 1Z1 Canada; 12 Department of Community Health & Epidemiology, University of Saskatchewan, Box 7, 107 Wiggins Road, Saskatoon, SK S7N 5E5 Canada; 13 Department of Medicine, University of British Columbia, Vancouver, BC Canada

**Keywords:** Juvenile idiopathic arthritis, Quality improvement, Quality Indicator

## Abstract

**Background:**

The study evaluates Performance Measures (PMs) for Juvenile Idiopathic Arthritis (JIA): The percentage of patients with new onset JIA with at least one visit to a pediatric rheumatologist in the first year of diagnosis (PM1); and the percentage of patients with JIA under rheumatology care seen in follow-up at least once per year (PM2).

**Methods:**

Validated JIA case ascertainment algorithms were used to identify cases from provincial health administrative databases in Manitoba, Canada in patients < 16 years between 01/04/2005 and 31/03/2015. PM1: Using a 3-year washout period, the percentage of incident JIA patients with ≥1 visit to a pediatric rheumatologist in the first year was calculated. For each fiscal year, the proportion of patients expected to be seen in follow-up who had a visit were calculated (PM2). The proportion of patients with gaps in care of > 12 and > 14 months between consecutive visits were also calculated.

**Results:**

One hundred ninety-four incident JIA cases were diagnosed between 01/04/2008 and 03/31/2015. The median age at diagnosis was 9.1 years and 71% were female. PM1: Across the years, 51–81% of JIA cases saw a pediatric rheumatologist within 1 year. PM2: Between 58 and 78% of patients were seen in yearly follow-up. Gaps > 12, and > 14, months were observed once during follow-up in 52, and 34%, of cases, and ≥ twice in 11, and 5%, respectively.

**Conclusions:**

Suboptimal access to pediatric rheumatologist care was observed which could lead to diagnostic and treatment delays and lack of consistent follow-up, potentially negatively impacting patient outcomes.

## Background

Juvenile Idiopathic Arthritis (JIA) is the most prevalent type of childhood inflammatory arthritis with a prevalence rate of between 3.8–400 per 100,000 in children under the age of 16 [1]. JIA represents a heterogeneous group of conditions associated with potentially damaging extra-articular manifestations including anterior uveitis. JIA is most commonly diagnosed and managed by pediatric rheumatologists, given their training and expertise [2–4]. Furthermore, many pediatric rheumatology centers in Canada offer a multidisciplinary approach to care, including physical therapy and occupational therapy along with medical management, to improve patient outcomes.

Delays in referral to a pediatric rheumatologist have been associated with greater disability and poorer quality of life [5]. Accordingly, current guidelines recommend early diagnosis and treatment of children and adolescents with JIA [4, 6, 7]. Waiting time benchmarks for JIA in Canada have been set at 4 weeks between referral and pediatric rheumatologist visit (with the exception of systemic onset JIA which is 7 days) [4, 8]. These benchmarks are similar to those set by other organizations including the British Society of Paediatric and Adolescent Rheumatology (BSPAR, referral within 10 weeks of symptom onset and visit 4 weeks from referral) [6]. Ongoing pediatric rheumatology care is also important, and recommended frequency of visits is based on a variety of factors, including disease severity and treatment monitoring [4].

The Arthritis Alliance of Canada (AAC) recently developed a set of System-Level Performance Measures for inflammatory arthritis, including rheumatoid arthritis, ankylosing spondylitis, psoriatic arthritis and JIA [8]. The 6 measures support early access to care, as well as ongoing subspecialty care and treatment for patients with inflammatory arthritis. The objective of this study was to evaluate the two AAC performance measures related to access to rheumatologist care and that are specifically applicable to JIA at a population-level: i) The percentage of patients with new onset JIA with at least one visit to a pediatric rheumatologist in the first year of diagnosis; ii) The percentage of patients with a diagnosis of JIA under the care of a pediatric rheumatologist seen in follow-up by a pediatric rheumatologist at least once per year.

## Methods

### Data sources

For the study, three databases from the Manitoba Population Research Data Repository housed at the Manitoba Centre for Health Policy (MCHP) were used: hospitalizations (the inpatient discharge abstract database, DAD), outpatient physician visits (practitioner billing claims), and the health insurance registry. The Repository maintains provincial administrative databases, which can be anonymously linked using a unique personal identifier, for the population of Manitoba, Canada, approximately 1.3 million people.

Ethics approval for the project was obtained from the University of Manitoba Health Research Ethics Board, approval number H2016:196(HS19755), and data access approval was provided by the Manitoba Health Information Privacy Committee. A waiver of consent was obtained for this study due to the use of deidentified data.

### Case definitions

A validated algorithm for JIA [9] was used to ascertain cases in the population < 16 years of age between April 1, 2005 and March 31st 2015. The algorithm contains codes for JIA but also for rheumatoid arthritis and ankylosing spondylitis, as in children under the age of 16, these likely represent categories of JIA. This algorithm was validated in the province of Manitoba against the gold standard of a rheumatologist’s clinical diagnosis and have a sensitivity of 89.2% (95% CI 86.8, 91.6), specificity of 86.3% (95% CI 83.0, 89.6), and a positive predictive value (PPV) of 90.6% (95% CI 88.3, 92.9) [9]. Cases were identified by either one DAD hospital separation with an International Classification of Disease (ICD)-10 CA code for JIA (M05.X, M06.X, M08.X, M45.X) or two or more physician billing claims (ICD-9-CM codes: 714.x or 720.x) for JIA ≥ 8 weeks apart but within 2 years [9].

### Calculation of performance measures

Two performance measures from the AAC System-Level Performance Measurement Set are applicable to JIA [8] and were operationalized in this study for health administrative data. The other performance measures in the set were not evaluated because they were either not applicable to JIA (i.e. 2 measures related to disease modifying treatment in rheumatoid arthritis) or could not be measured at a population level using administrative data (i.e. time to pediatric rheumatologist as referral dates are not captured in administrative data).

For the first performance measure, an incident cohort was used to ascertain the percentage of patients with new onset JIA with at least one visit to a pediatric arthritis specialist (see definition below) within the first year of diagnosis. To ascertain incident cases, a 3-year washout period was used to identify the first diagnostic code for JIA in the administrative data (April 1st 2005, March 31st 2008). Additionally, all cases had at least 6 months of health insurance coverage in the Manitoba population registry prior to the first diagnosis code. Cases meeting the performance measure had at least one code from a pediatric arthritis specialist within 365 days of their first JIA code.

The prevalent JIA cohort was used for the second performance measure to determine the percentage of JIA patients seen in annual follow-up. For the purpose of reporting this measure, once a patient is seen by a pediatric arthritis specialist at least twice, then that patient is considered to be under the care of a pediatric arthritis specialist until end of follow-up. The measure was computed by estimating, for each fiscal year between 2006 and 2015, the proportion of patients expected to be seen in follow-up during that period, who had a pediatric arthritis specialist visit (at a minimum one visit expected per fiscal year). Fiscal years extend from April 1 to March 31. The percentage of patients with gaps in care of > 12 and > 14 months between consecutive pediatric arthritis specialist visits was also calculated. The performance measure was originally described as the percentage of patients with JIA seen in yearly follow-up [8] as this minimum standard for care and is concordant with a current Canadian position statement on JIA care [4]. During further measure testing 2 additional issues were identified: 1) billing fees may increase if the patient is seen > 12 months between appointments; 2) additionally, some stable patients who are booked 1 year following their last appointment may get booked > 12 months from their last appointment due to patient and/or physician scheduling. Therefore, an extended observation period of 14 months was built into the measure to address these issues that may not be directly related to patient quality of care [10, 11].

There is no pediatric rheumatologist identifier in the administrative datasets in Manitoba because pediatric rheumatologists bill the provincial ministry of health on a fee-for-service basis as general pediatricians; therefore, an algorithm was created to identify pediatric arthritis specialists, including pediatric rheumatologists and pediatricians with a special interest in arthritis. A physician was identified as a pediatric arthritis specialist if he/she had a pediatrician identifier and at least 25 visits for individuals < 16 years in a year and at least 50% of visits in a year had a JIA diagnosis code. The algorithm aimed to capture all pediatric rheumatology locums in the province who practiced for at least 1 week. These locums were expected to see a minimum of 25 cases a week, based on a recent national rheumatology survey, which found that the median number of new patients and follow-up patients seen by pediatric rheumatologists per week was 4 (Interquartile range, IQR 2–5) and 15 (IQR 8–20) respectively [12]. The threshold for the number of rheumatology visits in a year with a diagnosis of JIA is based on pediatric registry studies demonstrating that between 30 and 60% of cases followed had a diagnosis of JIA [13, 14]. To ensure specificity of our algorithm we used a threshold of 50% (closer to the upper estimate of these studies). These criteria resulted in the identification of five physicians, which was concordant with known estimates of the numbers of pediatric arthritis specialists (including locums) in the province at the time. Once a physician met the criteria, he/she was considered a pediatric arthritis specialist for the remainder of the study.

## Results

Performance Measure 1: We identified 194 incident JIA cases diagnosed between April 1st 2008 and March 31st 2015. The median age at JIA diagnosis date was 9.1 years (Q1 5.5 and Q3 12.8) and 71% were female. Table 1 describes the percentage of JIA cases who saw a pediatric arthritis specialist within a year of diagnosis. Due to small numbers of cases (i.e., *n* < 7) in some reporting years, 2 year intervals were used for reporting. The percentage of JIA patients seen by a pediatric arthritis specialist within 1 year of their first diagnosis code ranged from 78 to 81% for all years except for the 2014/15 fiscal year, when it declined to 51%. This decline corresponded with a known decrease in access to pediatric rheumatologist services in the province.

**Table 1:** Incident JIA cases who saw a pediatric arthritis specialist within the first year after their first JIA diagnosis

Fiscal Year Period^a^	Diagnosed JIA incident cases	Percentage seen by a pediatric rheumatologist within a year
2008/2010	50	80
2010/2012	54	81
2012/2014	55	78
2014/2015	35	51

^a^Due to small cell sizes (*n* < 7) in some measurement years, reporting period was defined in 2-year intervals

**Fig. 1:**
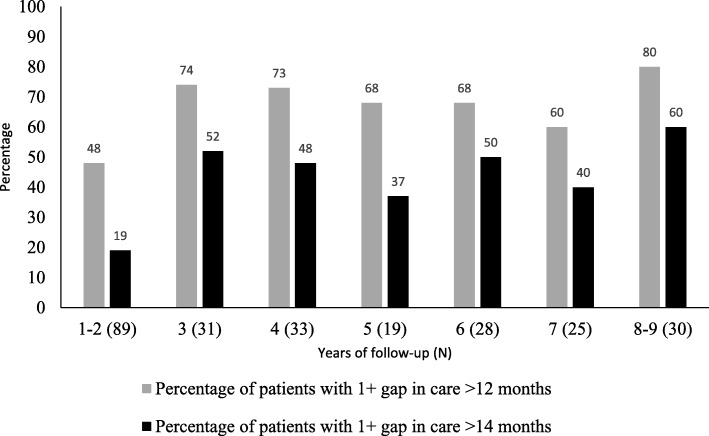
Percentage of patients with at least one gap in JIA rheumatology care by length of follow-up in years

Performance Measure 2: Table 2 summarizes the percentage of patients seen on an annual basis between 2006 and 2015. The decline in the percentage seen annually (83% seen in 2006/07 year to 58% seen in 2014/15) was not statistically significant (*p* = 0.47). Over half (52%) of all JIA patients experienced one gap and 11% experienced two or more gaps in care of > 12 months over the course of follow-up. This declined to 34% (1 gap) and 5% (2 or more gaps) if a 14-month window between consecutive visits was used. In Fig. 1, the proportions of patients with ≥1 gaps of > 12 or > 14 months in care are reported, stratified by the length of follow-up. The number of gaps in care increased with a longer duration of follow-up, and 80% of patients with 8 to 9 years of follow-up experienced at least one 12-month gap in care. This declined to 60% of patients who experienced one or more gaps in care when a 14-month interval was used.

**Table 2:** Observed and expected number of JIA follow-up visits by a pediatric arthritis specialist using fixed 12-month intervals

Fiscal year									
	2006/07	2007/08	2008/09	2009/10	2010/11	2011/12	2012/13	2013/14	2014/15
Observed	63	62	74	94	82	72	70	66	68
Expected	76	81	101	120	114	116	123	116	118
Percent^a^	83	77	73	78	72	62	57	57	58

^a^Percent refers to the percentage of patients seen each fiscal year. This is calculated based on the proportion of patients with a follow-up each fiscal year using the expected follow-ups (a minimum of 1 per fiscal year) as the denominator

### Discussion

This is the first study to formally evaluate the AAC system-level performance measures [8] in JIA (see also an abstract of this work presented at the Annual European Congress of Rheumatology [15]). Overall, 19 to 49% of patients with JIA were not seen by a pediatric arthritis specialist within 1 year of diagnosis, and half had at least one gap in care of > 12 months, while a third had at least one gap in care of > 14 months. While waiting times are not captured in administrative health data, the percentage of patients seen within a year offers an indication of the percentage of JIA cases who are not receiving care in keeping with national recommendations. Suboptimal access to rheumatologist care and lack of consistent rheumatologist care may lead to delays in treatment and ongoing care that could negatively impact patient outcomes.

Our study identified a number of patients diagnosed as JIA by a non-rheumatologist who did not see a pediatric arthritis specialist within a year of their diagnosis. Other physicians might have made the diagnosis of JIA, including general pediatricians, primary care practitioners, or pediatric orthopedic surgeons. The province of Manitoba is geographically large (area of 649, 950 KM^2^) and there is only one pediatric rheumatology center, therefore some patients may not be presenting to pediatric rheumatology for care in a timely fashion.

Long delays to pediatric rheumatology care have also been shown in Canadian children with newly diagnosed JIA from the Research on Arthritis in Canadian Children Emphasizing Outcomes (ReACChOut) cohort [16]. In this cohort 52 (16%) out of 319 patients enrolled in a study on waiting times did not see a pediatric rheumatologist for a year or more after symptom onset [16], and the median time from symptom onset to first pediatric rheumatologist visit was 115 days (interquartile (IQR) 45, 219 days). Heel pain or enthesitis were associated with delays to rheumatologist visit, while history of fever, a limp, higher parental education and South Asian ethnicity were associated with less delays [16].

Long delays to pediatric rheumatology care have also been found in other countries including Germany, where a study on wait times in JIA found a median referral time of 90 days (range 0–2, 160 days) [17], although the longest median time to referral was seen in patients with polyarticular-onset JIA (median 156.5 days, range 26–2160 days). In this study, referring physician subspecialty and distance from the pediatric rheumatology center were significantly associated with longer waiting times for care, namely patients seeing orthopedic surgeons prior to pediatric rheumatologist referral, and patients who lived ≥50 km from the pediatric rheumatology center had the longest delays to care [17].

Similarly, in the Childhood Arthritis Prospective Study (CAPS) from the United Kingdom, the median symptom duration at first presentation to the pediatric rheumatology center was 4.6 months (IQR 2.3, 9.5), and 108 (21%) of children had symptoms > 1 year [18]. Inflammation (measured by the ESR) was an independent predictor and children with a normal ESR had an odds of total symptom duration > 4 months before first appointment of 3.32 (95% CI 1.93, 5.69) [18]. The shortest wait times were seen in patients with systemic JIA, and the longest in psoriatic arthritis [18]. A follow-up study from CAPS evaluating waiting times and outcomes over the last 10 years found no improvements in access, with only 20% of the cohort seen within 10 weeks of symptom onset and up to one-third of children with new onset JIA still experiencing high disease activity at 1 year [19].

While the majority of studies on JIA waiting times are retrospective reviews or cohort studies, another approach has been the use of administrative data to examine this topic at a population level, similar to our study. A population-based study conducted in the Canadian province of Quebec between 1997 and 2003 identified 842 patients with at least one code for Juvenile Rheumatoid Arthritis (JRA) [20]. Only 66% of these suspected JRA cases had contact with an arthritis specialist during the study period, and 159 (45%) of patients diagnosed by a non-arthritis specialist (*n* = 352) visited an arthritis specialist over a subsequent 3-year follow-up [20]. Predictors of contact with an arthritis specialist included being female and living in an area with high service availability or if the arthritis was diagnosed by another specialist [20]. Arthritis specialists were also consulted sooner in younger patients [20]. Our study differed from this population-based study in that we used a more stringent and validated case-definition for JIA [9].

Few other studies have formally evaluated performance measures in JIA. In 2013, adherence to the BSPAR/Arthritis and Musculoskeletal Alliance (ARMA) Standards of Care for JIA was evaluated in the UK [21]. This evaluation required complex and detailed chart reviews at multiple centers. The results revealed delays in access to specialist care with only 41% seen ≤10 weeks from symptom onset and 60% seen ≤4 weeks from referral [21]. The BSPAR/ARMA Standards also recommend that follow-up appointments be scheduled at intervals of ≤4 months [6] and this standard was met 79% of the time [21], although the length of follow-up was shorter than in our study.

Our study has some limitations. While validated case definitions were used to identify JIA cases, it is possible that some of the cases that the algorithm identified as JIA were not JIA (false positives). However, if another physician type (primary care practitioner, general pediatrician or other) is recording JIA as the most responsible diagnosis multiple times as the reason for the visit on the billing claim sent to the provincial ministry, it is surprising that the patient isn’t always referred to a pediatric rheumatology center for confirmation of a diagnosis. Additionally, as the administrative data contain only the first 3 digits of an ICD code, we could not capture any JIA cases coded as psoriatic arthritis (which is specified using the fourth digit). There was also no additional information available on the category of JIA or disease activity, which may have impacted measured outcomes. In future studies linkage to registry data may provide useful additional information on the predictors of measure performance for health resource use planning (e.g., disease activity, clinical characteristics including subtype of JIA). The data also do not capture the date of symptom onset, and it is anticipated that further delays for some patients are likely not adequately represented in this study. Referral date was not captured in the administrative data, and the AAC performance measurement for waiting time could not be calculated. With increasing use of electronic medical records (EMRs) it is possible that waiting time to care could be captured using EMR data. In related work in RA models of care in Canada, linkage to triage databases was required to ascertain waiting times for rheumatologist care [22]. When considering the performance measures, it is important to remember that they represent minimal standards of care, and more frequent assessments are recommended to tailor treatment in patients with ongoing active disease [4].

Lastly, there was no pediatric rheumatologist identifier in the administrative dataset, so an algorithm was constructed based on known practice patterns of pediatric arthritis specialists in the province. While it is possible that this algorithm identified general pediatricians in addition to pediatric rheumatologists, this is unlikely given the threshold of 50% of billings for JIA. It is possible this algorithm underestimated the number of pediatric rheumatologists in the province; however, the number of practitioners identified with the algorithm was aligned with known numbers of pediatric rheumatologists (including locums) in the province, which provided face validity to the methodology. The challenges of identifying rheumatologists using Canadian administrative data is not unique to this study. In other provinces additional databases have been developed to better identify rheumatologists for epidemiologic research including in Ontario [23]. In future developing a database of provincial rheumatologists (pediatric and adult) may be of use for epidemiologic studies.

## Conclusions

In conclusion, the study demonstrates lower than expected consultations with a pediatric rheumatologist for children and adolescents with JIA in Manitoba, and delays in follow-up for many patients under rheumatology care. It is important that even suspected JIA cases should be referred to a pediatric arthritis specialist for confirmation of diagnosis and initiation of treatment to avoid potentially debilitating outcomes including erosions, flexion contractures, growth abnormalities and vision loss due to uveitis. In future, studies evaluating the predictors of measure performance should be conducted to identify targets for interventions to improve performance. Further study on the impacts of these care gaps on patient outcomes is also warranted.

## Data Availability

The data that support the findings of this study are available from the Manitoba Centre for Health Policy but restrictions apply to the availability of these data, which were used under license for the current study, and so are not publicly available. Details on how to access to the repository are found on the following website: http://umanitoba.ca/faculties/health_sciences/medicine/units/chs/departmental_units/mchp/resources/repository/index.html

## Abbreviations

AAC: Arthritis Alliance of Canada
ARMA: Arthritis and Musculoskeletal Alliance
BSPAR: British Society of paediatric and adolescent rheumatology
CAPS: Childhood Arthritis Prospective Study
DAD: Discharge Abstract Database
ICD: International Classification of Diseases
JIA: Juvenile Idiopathic Arthritis
JRA: Juvenile Rheumatoid Arthritis
MCHP: Manitoba Centre for Health Policy
ReACChOut: Research on Arthritis in Canadian Children Emphasizing Outcomes
UK: United Kingdom
